# A new approach for reducing pollutants level: a longitudinal cohort study of physical exercises in young people

**DOI:** 10.1186/s12889-022-12621-2

**Published:** 2022-02-03

**Authors:** Yujuan Xu, Hongliang Gao, Zhixiang Du, He Liu, Qi Cheng, Furong Zhang, Juan Ye, Aiqing Wang, Yanjun Dou, Bei Ma, Ningwei Zhao, Feng Zhu, Xianlin Xu, Ning Shen, Jing Wu, Bin Xue

**Affiliations:** 1 Hohai University, Nanjing, 210098 China; 2 Core Laboratory, Sir Run Run Hospital, Nanjing Medical University, Nanjing, 211166 China; 3 Department of Infectious Diseases, Taizhou People’s Hospital, Taizhou, 225300 China; 4 General surgery department, Sir Run Run Hospital, Nanjing Medical University, Nanjing, 211166 China; 5 Medical School of Nanjing University, Nanjing, 210093 China; 6 Affiliated Hospital of Integrated Traditional Chinese and Western Medicine, Nanjing University of Chinese Medicine, Nanjing, 210028 China; 7 Affiliated Hospital of Nanjing University of Chinese Medicine, Nanjing, 210029 China; 8 Department of Urology, Sir Run Run Hospital, Nanjing Medical University, 109 Longmian Road, Jiangning, Nanjing, 211100 Jiangsu China; 9 China Exposomics Institute (CEI) Precision Medicine Co. Ltd, Shanghai, 200120 China

**Keywords:** Environmental exposure, Physical exercise, Elimination of pollutants, Dimethoate, BaP, BPA

## Abstract

**Background:**

The present study aimed to evaluate the elimination of three common pollutants (dimethoate, benzo(a)pyrene (BaP) and bisphenol A (BPA) by different physical exercises and to assess the possible factors which could affect the pollutants elimination.

**Methods:**

A total of 200 individuals who chose different kinds of exercises in accordance to their own wish were recruited. The levels of urinary pollutants were measured using β-glucuronidase hydrolysis followed by a high-performance liquid chromatography tandem mass spectrometry-based method.

**Results:**

Totally, the levels of dimethoate, BaP and BPA were reduced after physical exercises. However, the elimination of BaP in male was higher than that in female but the elimination of BPA in female was higher than that in male. Multivariate logistic regression showed that the degree of heart rate (HR) change was a protective factor affecting the improvement effect of dimethoate, BaP and BPA while BMI (body mass index) was a risk factor. Nevertheless, sex was a risk factor affecting the improvement of dimethoate and BaP but had a lower efficacy on BPA improvement.

**Conclusion:**

The present findings indicate that physical exercises can be considered as a novel approach to eliminate pollutants level in human body and can also give suggestions for choosing specific physical exercises to male and female individuals. Moreover, those who are with higher BMI need to lose weight before eliminating pollutant level through physical exercises.

## Introduction

Excessive accumulation of environment pollutants in tissues and organs tends to cause damage to the human body, eventually leading to the various diseases. Due to global climate change, pesticides abuse and industry development, individuals are frequently exposed to pollutants from mining and smelting metal ores, pesticide manufacturing and application, wood preservatives, even drinking and food intake [1, 2]. There are three common environmentally-derived pollutants that require special attention, which are organophosphorus pesticide residues, dimethoate [3], from pesticide abuse, polycyclic aromatic hydrocarbons(PAHs), benzo(a)pyrene (BaP) [4], from air pollution, and bisphenol A(BPA) [5] from the plastics industry.

Dimethoate, an organophosphorus insecticide, is widely used for controlling a wide range of insects. However, many studies have shown adverse effects on mammalian [6–8] . Several studies have documented that the carcinogenicity [9] and developmental toxicity [10, 11] of dimethoate which can lead to a reduction in plasma cholinesterase [12] . Likewise, BaP has been characterized as a stronger carcinogen than other PAHs compounds [13] which can cause DNA damage and plays a role in lung carcinogenesis [14], atherosclerosis [15] and so on [16–18]. BPA is an endocrine disruptor chemical which can induce carcinogenesis, reproductive toxicity, abnormal immune response, and developmental disorders of brain and nervous system through various signaling pathways [19] .

More recently, there has been growing recognition of the vital links between pollutants accumulation and health. Different approaches for these pollutants removal from the environment including biodegradation and non-biodegradation have been developed so far [20] . Well-known methods include degradation (chemical or microbial) or adsorption on different types of materials [21] . However, the elimination of the pollutants that have already been abnormally accumulated in human body has not been reported**.**

It has been reported that physical exercise is an efficient non-pharmacological intervention for human health [22, 23]. Exercise has also been shown to be beneficial in the treatment of many chronic diseases through changing the metabolism state, strengthening muscle, and improving immunity [24] . Physical exercise is considered a part of healthy life style. The individuals who exercise regularly can expect positive feedback from their environment and social contact, and even can be beneficial for the treatment of psychiatric diseases [25]. Marisa Toups [26] evaluated the effect of exercise for depression patients during 12 weeks of exercise augmentation and found positive valence symptoms improvement with exercise treatment for depression, and this change correlates well with overall outcome. Likewise, the improvement of metabolic diseases like obesity, hyperlipidemia, metabolic syndrome, polycystic ovarian syndrome, type 2 diabetes, type 1 diabetes by exercise had also been reported [27] . In a clinical study, post-dinner moderate-intensity exercise of those patients with type 2 diabetes could reduce the 2-hour postprandial glucose spike, mean glucose level, and peak glucose level compared to the control condition [28] .Moreover, regular physical exercises could induce antiatherogenic adaptations in vascular function and structure, improve myocardial regeneration capacity and cardiac parasympathetic regulation [29] . Besides, physical exercises can also improve lung function [30] and the quality of life in patients with malignant tumor [31] . Exercise could also reduce the likelihood of air pollution-related mortality [32, 33]. The extent, however, to which elimination of environment pollutants may be attributed to physical exercises is still poorly understood.

Here, we recruited 200 individuals to receive a physical exercise for a period of 3 months in order to assess the correlation between the improvement effect of pollutants and different physical exercises. What’s more, identification of the most significant factors which can influence the improvement of physical exercise intervention may provide new insights into pollutants elimination in young people.

## Materials & methods

### Study design and participants

All procedures including sampling and examination were performed in agreement with the principles set in the Declaration of Helsinki and its later amendments (2013). All participants were informed about the objectives of the study and experimental procedures and signed the informed consent form. A total of 200 freshmen of the Hohai University (Nanjing, Jiangsu province, China) were analyzed, in which there were 100 males and 100 females aged from 17 to 19. The height, weight, BMI, and changes in urine pollutants (dimethoate, bisphenol A, Benzo(a)pyrene) of each participant before and after the exercise program were recorded.

### Inclusion and exclusion criteria

Inclusion criteria: a) Top 100 male and female with higher pollutants level [34]; b) Aged from 17 to 19; c) Did not engage in any regular exercise activities for 2 months prior to enrollment in the study (Regular exercise was defined as exercising more than once a week.).

Exclusion criteria: a) Unable to participate in the exercise due to physical disability; b) Severe heart, lung, liver, or renal disorders; c) Participating in other exercise after choosing the exercise according to preference;

### Exercise program

The exercise program lasted 3 months, 3 times a week, 90 min every time, the subjects were not allowed to participate in any other exercises after they chose their preference exercise in their leisure Duration. Exercise items include basketball, volleyball, ping pong, handball, tennis, football, badminton, Latin dance, rhythmic gymnastics, bodybuilding, yoga, and shaping. Subjects from Hohai University underwent 90 min sessions. The heart rate (HR) before and after exercise was monitored. The first 30 min of exercise were a warm-up period, where speed was progressively increased and aerobic exercise with moderate severity equal to 60–70% of the maximum HR. Peak heart rate will be recorded immediately upon the end of the exercise. Thereafter, 10 min of cooling down consisted of stretching movements of muscles in lower extremities and 1-min relaxation of the whole body.

### Sample collection

Collection of morning urine samples (second portion) was performed using plastic Vacuette® Urine Collection Cups (Greiner Bio-One International AG, Austria). Only healthy subjects without chronic diseases were involved in the current investigation in order to avoid side effects and interactions of diseases on the studied parameters.

### Sample processing

Samples were collected at predetermined time intervals and filtered through a membrane filter of 0.22 μm. The pH of respective mixtures was adjusted to 5.4 by adding acetic acid-sodium acetate buffer (0.5 M), then β-glucuronidase/arylsulfase (10 μL), and vitamin C (5 mg) were added and incubated overnight at room temperature to complete the enzymatic hydrolysis. The samples after enzymatic hydrolysis were extracted by solid-phase extraction with an SPE column (C18 ENVIJI 0.25 g). The extract was eluted with methanol (2 mL) and dried with nitrogen. Finally, methanol (100 μL) was re-dissolved as the analyte to be determined. 50 μL of the analyte to be tested was transferred to a liquid chromatography bottle with a microsyringe, which was specially used for the injection analysis of BaP. Evaluation of dimethoate, BPA, and BaP levels in the urine of examinees was performed using Liquid chromatography-mass spectrometry (LC-MS). The 1 μg/ml standard working solutions of dimethoate, BPA, and BaP were prepared with methanol as solvent. After continuous dilution of 104 times, it became the standard working solution of 100 pg/ml. Take BaP as an example, different concentrations of BaP standard working solution were prepared. 50 μL of each BaP standard working solution was transferred to a liquid chromatography bottle with a microsyringe, which was specially used for the injection analysis of BaP standard samples.

### Statistical analysis

The data were expressed as the frequencies (n), percentages (%), means ± standard deviations (SDs), median (inter-quartile range), and were analyzed with R version 4.0.2. Count data were analyzed with the chi-square test. If measurement data conformed to a normal distribution, the t-test was used to analyze. The Mann-Whitney U-test was used for comparing unorthodox distribution data. Before regression analysis, the factors are verified by multiple linear regression to verify independence. The difference value before and after exercise was calculated and categorized into three grades. The possible effects of the determinants on three pollutants were analyzed through multivariate logistic regression analysis. A difference with *P* < 0.05 was considered statistically significant.

## Results

### Characteristics of individuals

A total of 200 individuals were enrolled in the present study (Table 1), including 100 female and 100 male individuals. Participants can choose preference exercise activities depending on their own interests. Ultimately, 12 kinds of sports were included in the final cohort (basketball, volleyball, ping pong, handball, tennis, football, badminton, Latin dance, rhythmic gymnastics, bodybuilding, yoga and shaping).

**Table 1:** Characteristics of individuals

Values	Male(n%)		Female(n%)		Total(n%)		Statistic(t/χ^**2**^)	***P-Value***
**n**	100		100					
**Height**(cm)	174.98 ± 5.49		162.27 ± 4.49		168.62 ± 8.10			
**weight (**kg)	65.6 ± 6.06		54.66 ± 4.89		60.13 ± 7.76			
**Waistline(**cm)	76.71 ± 5.08		69.85 ± 4.63		73.28 ± 5.94			
**BMI** (kg/m^2^)	21.41 ± 1.46		20.74 ± 1.36		21.07 ± 1.45			
**Sport type**								
Basketball	17	10.00%	0	0.00%	17	17.00%		
Volleyball	0	0.00%	16	14.00%	16	16.00%		
Ping pong	9	9.00%	32	32.00%	41	41.00%		
Handball	0	0.00%	12	12.00%	12	12.00%		
Tennis	22	22.00%	0	0.00%	22	22.00%		
Football	18	18.00%	0	0.00%	18	18.00%		
Badminton	24	24.00%	13	13.00%	37	37.00%		
Latin dance	0	0.00%	3	3.00%	3	3.00%		
Rhythmic gymnastics	0	0.00%	5	5.00%	5	5.00%		
Bodybuilding	10	10.00%	0	0.00%	10	10.00%		
Yoga	0	0.00%	6	4.00%	6	6.00%		
Shaping	0	0.00%	13	13.00%	13	13.00%		
RHR	82.50 ± 10.76		81.86 ± 10.32		82.18 ± 10.52		0.429	0.640
PHR	130.63 ± 22.69		131.46 ± 21.67		131.05 ± 22.14		0.729	0.830
HR(D-value)	48.13 ± 20.18		49.60 ± 20.36		48.87 ± 20.23		0.609	1.470

*Note:* Statistical methods: Independent sample T test, chi-square test. RHR:resting heart rate; PHR:postexercise heart rate; HR(D-value) = PHR-RHR

### Levels of three environmental pollutants were reduced after physical exercises

**Fig. 1:**
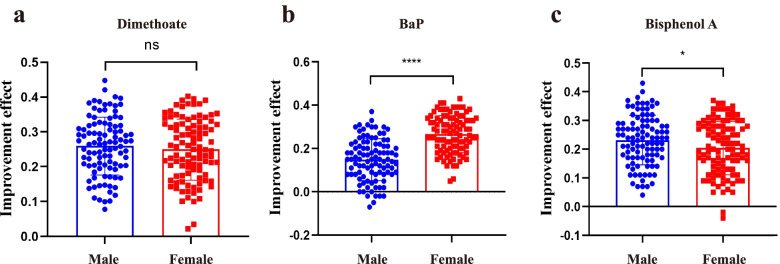
Comparation of the elimination of three pollutants between male and female. **a**:The improvement effect on dimethoate between male &female; **b **The improvement effect on BaP between male &female; **c** The improvement effect on BPA between male &female). *Note:* Improvement effect = (post-intervention level minus pre-intervention level)/post-intervention level

The level of dimethoate, BaP and BPA of 200 individuals were measured before and after 3 months physical exercises by a high-performance liquid chromatography tandem mass spectrometry-based method. Totally, the level of dimethoate, BaP and BPA (8.06 ± 1.51 ng/mL,0.32 ± 0.08 pg/mL,3.19 ± 0.54 ng/mL) were lower than that (10.78 ± 1.37 ng/mL, 0.4 ± 0.05 pg/mL, 4.07 ± 0.38 ng/mL) after physical exercises, respectively, of which the differences were statistically significant (Table 2). Besides, effects of gender on pollutants elimination were analyzed. There is no statistical difference on the elimination of dimethoate between male and female (Fig. 1a) The elimination of BaP in female was higher than that in male (Fig. 1b). However, the elimination on BPA in male was higher than that in female (Fig. 1c).

**Table 2:** Comparation of the level of three pollutants before and after exercise

Values	Before-exercises			After-exercises			Statistic(t)	P-Value
	Male (100)	Female (100)	Total	Male (100)	Female (100)	Total		
**Dimethoate** (ng/mL)	11.82 ± 1.05	9.74 ± 0.68	10.78 ± 1.37	8.79 ± 1.45	7.34 ± 1.18	8.06 ± 1.51	42.101	0.000^#^
**BaP** (pg/mL)	0.44 ± 0.04	0.36 ± 0.03	0.4 ± 0.05	0.38 ± 0.06	0.27 ± 0.04	0.32 ± 0.08	29.084	0.000^#^
**BPA** (ng/mL)	4.03 + 0.34	4.12 ± 0.39	4.07 ± 0.38	3.10 + 0.51	3.28 ± 0.55	3.19 ± 0.54	35.228	0.000^#^

*Note:* Statistical methods: Paired Sample T test;

^#^: Comparation between total before-exercises of pollutants level and after-exercises of pollutants level. 0.000:*P* < 0.0001

### The effect of gender preference physical exercises on the elimination of pollutants

**Fig. 2:**
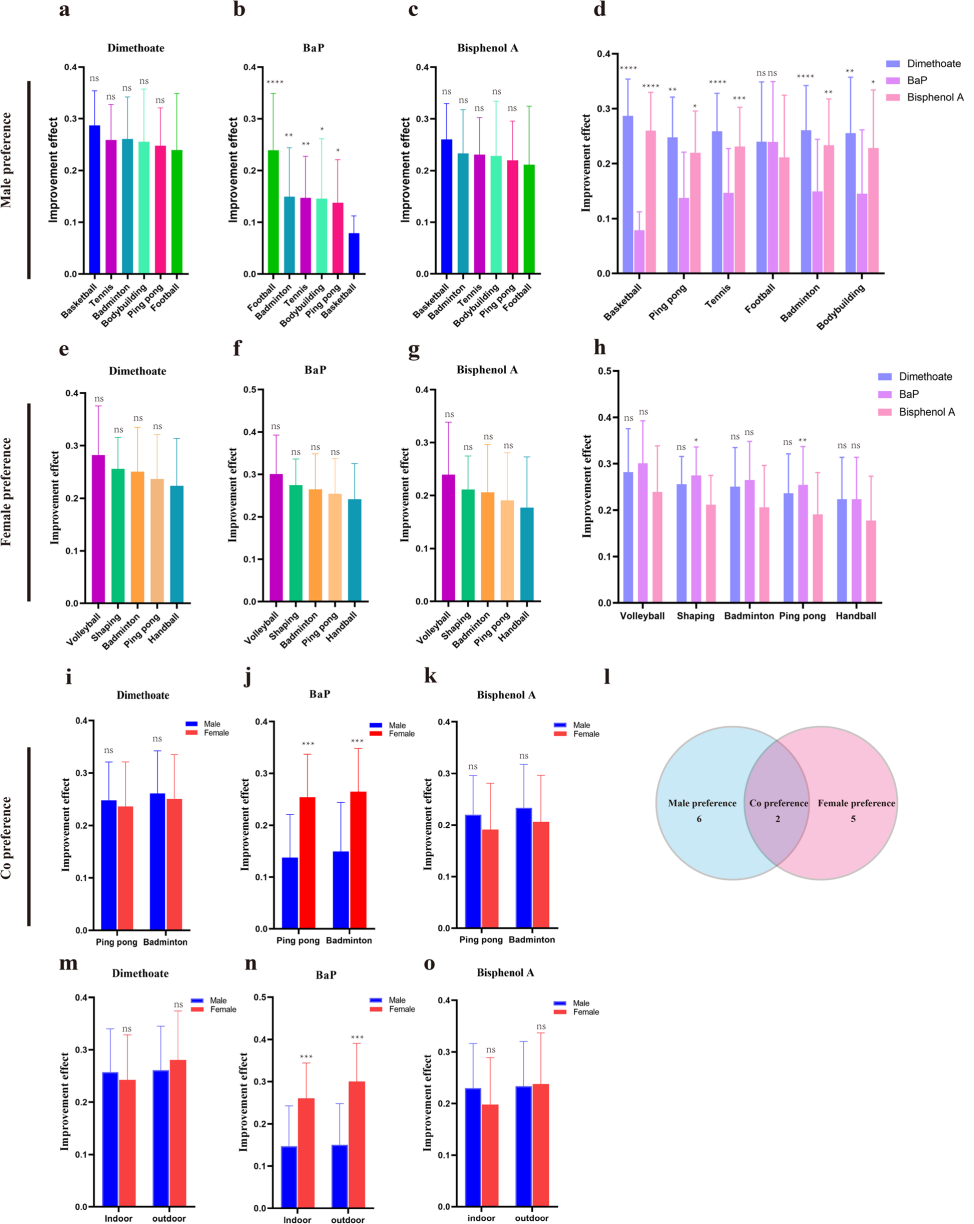
Elimination potential of different physical exercises on three pollutants. (**a**-**d**: Elimination potential of different physical exercises on three pollutants in male preference physical exercises; **e**-**h**: Elimination potential of different physical exercises on three pollutants in female preference physical exercises; **i**-**k**: Elimination potential of different physical exercises on three pollutants in co preference physical exercises; **l**: Definition of male preference physical exercises, female preference physical exercises and co preference physical exercises; **m**-**o**:Elimination potential of indoor and outdoor physical exercises on three pollutants)

Due to the gender difference, men and women have preferences when choosing physical exercise. In order to perform statistical analysis, we selected those physical exercises with subjects more than 8 to analyze among the 12 sports. Although there was no statistical difference on the improvement effect between football and other male preference exercises, basketball showed the best elimination potential (Fig. 2a, c) and football showed the worst elimination potential on dimethoate and BPA. Nevertheless, football revealed the best elimination potential on BaP (Fig. 2b). All male preference physical exercises showed the best elimination potential on dimethoate but the worst on BaP (Fig. 2d). Interestingly, all 5 female preference physical exercises showed the same tendency of elimination potential on three pollutants but there was no statistical difference (Fig. 2e-g) Conversely, all female preference physical exercises showed the best elimination potential on BaP (Fig. 2h). And in co preference exercises, the same elimination potential was observed which showed the best elimination potential on BaP (Fig. 2j) but no difference was observed on dimethoate and BPA (Fig. 2i,k). Male preference physical exercises, female preference physical exercises and co preference physical exercises were defined according to gender preference (Fig. 2l).Furthermore,we splitted the 12 sports into indoor (Ping pong, handball, badminton, Latin dance, rhythmic gymnastics, bodybuilding, yoga and shaping) and outdoor exercises (Basketball, volleyball,tennis, football) and investigated the elimination potential between genders on the three pollutants. The results showed no statistical difference on the improvement effect between male and female on dimethoate and BPA (Fig. 2m,n) but female seemed to have a better pollutant level reduction on Bap in both indoor and outdoor exercises (Fig. 2o).

### Linear correlation verification of difference factors

**Fig. 3:**
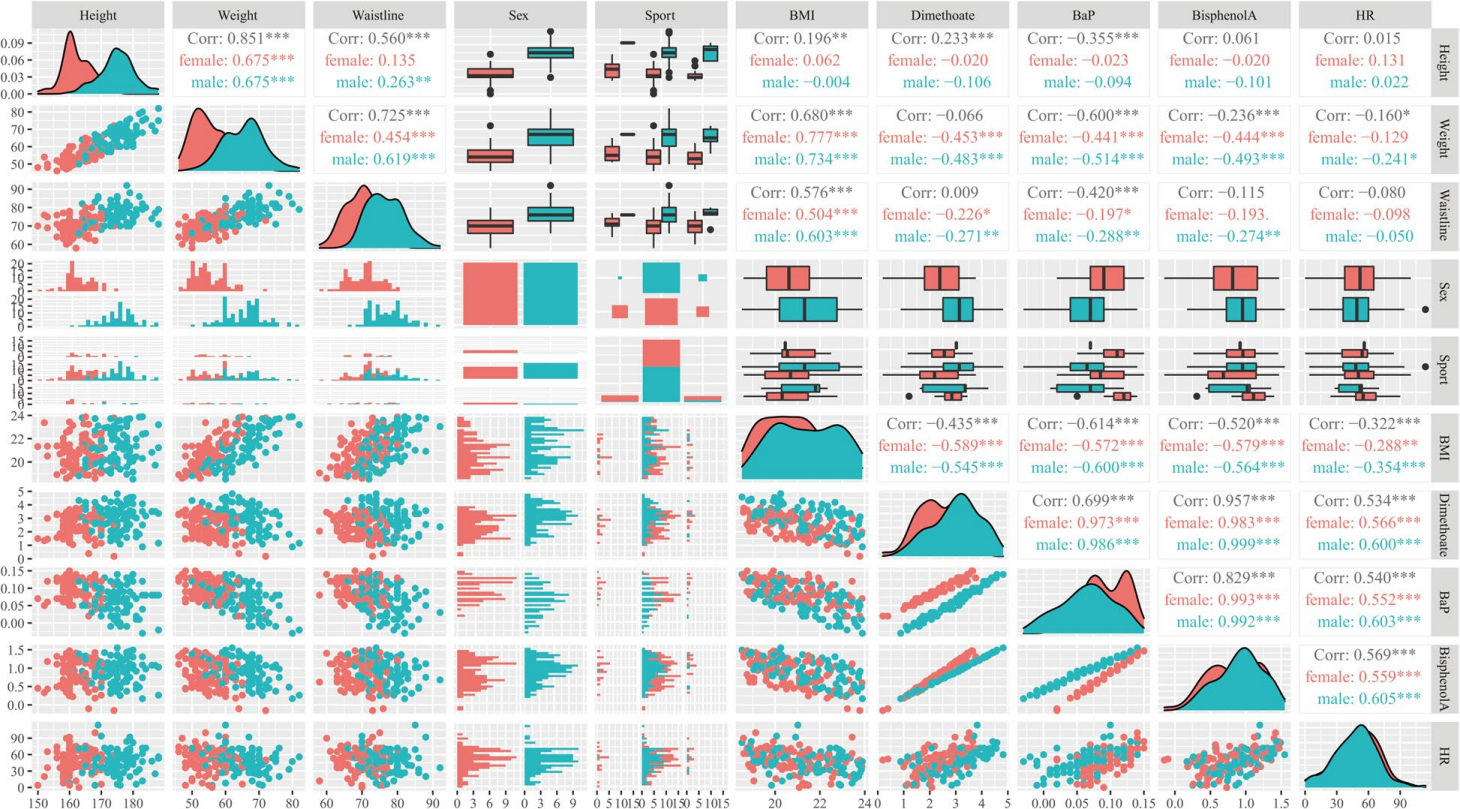
Linear correlation verification of difference factors. *Note:* The Spearman rank correlation coefficient validated the positive correlation among various factors,the red point in the figure represents BMI,height,weight, HR,level of dimethoate, BaP and bisphenol A after exercise-intervention of female repectively and the blue point represents these indicators of male

The difference factors were verified by linear correlation: in male and female participants, there was no significant correlation among the difference values of height, weight, waistline, dimethoate, BaP, BPA and HR. There was a high correlation between BMI and the level of dimethoate, BaP and BPA after exercise-intervention. BMI was highly correlated with height、weight、waistline, however, sport type and HR were not significantly associated with the rest indicator (Fig. 3).

### Multivariate ordered logistic and logistic regression analysis

Table 3 presents the predictive factors towards the different outcomes of interest using multivariate ordered logistic and logistic regression analysis. The results showed that the degree of HR change was the risk factor influencing the elimination of dimethoate, BaP and BPA, and the influence degree of the three pollutants was equal. BMI was a protective factor influencing the elimination of dimethoate, BaP and BPA. Those who are with lower BMI, the elimination of the pollutants was more obvious, and the difference was statistically significant. Gender also affects the elimination of these pollutants. But the type of physical exercise had no significant effect on the three pollutants elimination.

**Table 3:** Multivariate ordered logistic and logistic regression analysis

Factors		***β***	***S.E***	***Wald***	***P-Value***	OR	95%CI (lower-uper)	
**Dimethoate**	HR(D-value)	0.063	0.009	50.765	0.000	1.065	1.046	1.083
	BMI	− 0.585	0.109	28.669	0.000	0.557	0.450	0.690
	Sex	1.211	0.307	15.564	0.000	3.356	1.839	6.124
	Sport type	0.459	0.440	1.090	0.296	1.583	0.668	3.750
**BaP**	HR(D-value)	0.069	0.009	55.957	0.000	1.071	1.052	1.091
	BMI	−0.729	0.115	40.378	0.000	0.482	0.385	0.604
	Sex	0.947	0.307	9.488	0.002	2.578	1.411	4.708
	Sport type	0.258	0.445	0.336	0.562	1.294	0.541	3.093
**BPA**	HR(D-value)	0.060	0.009	45.206	0.000	1.061	1.043	1.080
	BMI	−0.882	0.126	49.306	0.000	0.414	0.323	0.529
	Sex	−2.487	0.348	51.134	0.000	0.083	0.042	0.164
	Sport type	0.479	0.458	1.093	0.296	1.614	0.658	3.963

*Note:* The Sex variable was assigned to male

## Discussion

Excessive accumulation of pollutants in human body could lead to the morbidity of various diseases. However, the possible harms of these pollutants are tended to be ignored [35] . Moreover, because of abnormal concentrations of pollutants on health, it is urgent to find ways to reduce the pollutants levels in the human body. Current approaches to remove common environment pollutants are based on the membrane technology for drinking water and reducing the source of production. Nevertheless, how to eliminate the pollutants that had already been accumulated in the human body is still underexplored and underreported. The notion, “Exercise as medicine”, has been termed as exercise and physical activity are increasingly becoming key tools in the treatment and prevention of several medical conditions including arthritis and diabetes [36] and more importantly physical exercise could serve as a tool to help the immune system against COVID-19 [37] . The beneficial adaptive responses to regular physical exercise are appreciated and include improvements in metabolism, redox state, and inflammation scenario in several tissue [38]. Moreover, several studies had indicated that regulate physical exercises could reduce the air pollution-related mortality and suggested that aerobic exercise training protects against air pollution and environmental tobacco smoke-induced lung inflammation [32, 33, 39, 40]. But to our knowledge, this is the first report about elimination of pollutants by physical exercises Therefore, we aim to determine if physical exercises had a positive effect on the elimination of various pollutants.

Our previous study showed that the average level of environmental pollutants accumulation in young people is relatively high and differs in the youth who live in different regions [34]. The young individuals seem to have relatively irregular diets and lifestyles which could increase the risk of pollutants accumulation. Herein, we reported that the levels of dimethoate, BaP and BPA were significantly reduced after 3 months’ physical exercises in young people.

The result revealed that exercise could serve as an efficient way to eliminate the pollutants level in the young people. Besides, the elimination potential appears to differ by sex. The elimination potential on BPA in male was higher than that in female; Inversely, the elimination potential on BaP in female was higher than that in male. Interestingly, the level of dimethoate and BaP in male was significantly higher than that in female before exercise which may suggested that different lifestyle like smoking and eating barbecued food could contribute to the accumulation of BaP [41, 42].

Different exercises seem to have various elimination potential on environmental pollutants in the present study. Take the male preference physical exercises as an example, basketball showed the best elimination potential on dimethoate and BPA, although there was no statistical difference on the elimination potential among all male preference physical exercises. This provides a very good reference for male when choosing exercises. But due to insufficient samples size of each physical exercise,no statistically significant difference was obtained among the elimination potential of different male preference physical exercises and female preference physical exercises on dimethoate and BPA. One unexpected result was that all male preference physical exercises showed the worst elimination potential on BaP among three pollutants, however, the results in female preference physical exercises were opposite. The same results were observed in indoor exercise and outdoor exercises. It is reported that BaP, one of the common PAHs, has been known as a kind of xenoestrogen, whose pollutants possess estrogenic and/or antiestrogenic activities [43]. This may contribute to the difference in the elimination potential on BaP between men and women and may also indicate that the metabolism of BaP is dependent on estrogen but the exact mechanism remains to be explored.

Since the lipophilicity of most persistent organic pollutants (POPs), it has been widely studied that POPs could be stored in the adipose tissue. Herein, we found that BMI had a high correlation with the elimination potential on dimethoate, BaP and BPA after exercise-intervention. In addition, multivariate ordered logistic revealed that those who are with lower BMI tended to have better elimination of pollutants. This rather interesting finding might be explained by the fact that some obese individuals with metabolic disorder may experience the abnormal metabolism of pollutants [44, 45] which means that a normal BMI is required for a better elimination of pollutants. Besides of the index of BMI, we also found that HR change was a risk factor influencing the elimination of dimethoate, BaP and BPA. It was well documented that alteration of HR could reveal the intensity of exercise [46]. This indicated that a certain intensity of exercise is needed to achieve a better elimination of pollutants.

Taken together, these findings suggest that physical exercise is a novel and efficacious approach for reducing pollutants level in young people and could contribute to our understanding of the health benefits of physical exercises and could partially provide a reference on promoting the young individuals to actively participate in physical exercises which have the best elimination potential on pollutants, thus develop the habit of regular physical exercise, and improve self-health ability and physical health level. But due to insufficient samples size of each physical exercise,we did not obtain a satifying results that the elimination potential of different physical exercises on the three pollutants. Therefore,it is necessary to further expand the sample size in the follow-up studies due to the small sample size and the absence of longterm follow-up studies included in this study.

## Conclusion

The present findings indicate that physical exercises can be considered as a novel approach to eliminate pollutants level in human body and can also give suggestions for choosing specific physical exercises to male and female individuals. Moreover, those who are with higher BMI need to lose weight before eliminating pollutant level through physical exercises.

## Data Availability

All data generated or analyzed during this study are included in this published article.

## Abbreviations

BaP: Benzo(a)pyrene
BPA: Bisphenol A
BMI: Body mass index
PAHs: Polycyclic aromatic hydrocarbons
LC-MS: Liquid chromatography-mass spectrometry
DNA: Deoxyribonucleic acid
HR: Heart rate
RHR: resting heart rate
PHR: Postexercise heart rate
POPs: Persistent organic pollutants
